# Near Infrared Fluorescent Nanostructure Design for Organic/Inorganic Hybrid System

**DOI:** 10.3390/biomedicines9111583

**Published:** 2021-10-30

**Authors:** Kyohei Okubo, Masakazu Umezawa, Kohei Soga

**Affiliations:** Department of Materials Science and Technology, Tokyo University of Science, Tokyo 125-8585, Japan; masa-ume@rs.tus.ac.jp (M.U.); mail@ksoga.com (K.S.)

**Keywords:** near infrared light, fluorescence, bioimaging, nanoparticles, hybrid nanostructure, polarity, phonon engineering

## Abstract

Near infrared (NIR) light offers high transparency in biological tissue. Recent advances in NIR fluorophores including organic dyes and lanthanide-doped inorganic nanoparticles have realized the effective use of the NIR optical window for in vivo bioimaging and photodynamic therapy. The narrow energy level intervals used for electronic transition that involves NIR light, however, give rise to a need for guidelines for reducing heat emission in luminescence systems, especially in the development of organic/inorganic hybrid structures. This review presents an approach for employing the polarity and vibrational energy of ions and molecules that surround the luminescence centers for the development of such hybrid nanostructures. Multiphonon relaxation theory, formulated for dealing with heat release in ionic solids, is applied to describe the vibrational energy in organic or molecular systems, referred to as phonon in this review, and we conclude that surrounding the luminescence centers either with ions with low vibrational energy or molecules with small chemical polarity is the key to bright luminescence. NIR photoexcited phosphors and nanostructures in organic/inorganic mixed systems, designed based on the guidelines, for photodynamic therapy are reviewed.

## 1. Design Basics of Near Infrared Fluorescent Nanostructure

Light in the near infrared (NIR) wavelength region exhibits high optical transmittance to living organisms and has been attracting increasing attention for applications in biomedical photonics. With a specific focus on this region ever since 2005, the authors have been developing NIR-emitting probes for biomedical photonics, such as fluorescence bioimaging, photodynamic therapy, and thermometry [1]. NIR light, as a photon, has a smaller amount of energy than that in visible light, resulting in narrow energy separations between an excited state and a ground state in optical transitions of the luminescence systems. Excitation energy in such systems is eventually converted into the form of either light or heat. Therefore, suppressing heat emission is of utmost importance in the rational design of phosphors that involve NIR light.

For bioimaging, organic dyes, quantum dot-, carbon nanotube-, and trivalent lanthanide (Ln^3+^)-containing inorganic nanoparticles are the major phosphors that emit NIR light [1]. Our recent review on the design of fluorescent materials with Ln^3+^ ions discussed the thermal interaction between Ln^3+^ ions, as luminescence centers, and surrounding atoms, ions, and molecules [2]. Thermal release of Ln^3+^ ions in ionic crystals has been semi-quantitatively formulated since the 1960s and is described as multiphonon relaxation theory. Even though the term “phonon” was originally defined in crystals, here for molecules, we will also refer to the “thermal quantum of vibration” as “phonon”. For ease of explanation, let us consider the case where there is only one type of phonon whose energy is ℏω, and when an electronic transition occurs by emitting or absorbing phonons, only one type of phonon is emitted. If the probability of absorption is P, and the number of required phonons is *n*, the probability (rate) can be written as $P^{n}$. Furthermore, n can be written as n=∆E/ℏω, where the energy separation for the electronic transition is ∆E [3,4]. The transition rate of multiphonon relaxation is given as

(1) $$\begin{array}{l} W_{\mathrm{MPR}}\left(T\right)=W_{\mathrm{MPR}}\left(0\right){\left[1-\exp\left(-\frac{∆E}{k_{B}T}\right)\right]}^{-n}\\ =W_{\mathrm{MPR}}\left(0\right){\left[1-\exp\left(-\frac{∆E}{k_{B}T}\right)\right]}^{-\frac{∆E}{\hbar \omega }}. \end{array}$$

Simply at a constant temperature, the equation

(2) $$W_{\mathrm{MPR}}=C\exp\left(-\frac{∆E}{\hbar \omega }\right),$$

has also been conveniently and often used [5]. These equations tell us that the smaller the ∆*E*, the smaller the number of phonons that can cause the transition for the phonon absorption and emission, and the easier it is for heat absorption and emission. Therefore, compared with the design for obtaining fluorescence of ultraviolet light and visible light, phosphors that emit NIR light and systems that are excited by NIR light are required to have a more delicate design for emitting light by preventing heat release.

Most discussions on the multiphonon relaxation of Ln^3+^ ions in crystals have been related to the relaxation rates to temperature, ∆E and ℏω [6]. $W_{\mathrm{MPR}}\left(0\right)$ in Equation (1) and C in Equation (2) include electron–phonon coupling strength; however, these constants have been overlooked, and very few documents have formally described the coupling strength in substances. Furthermore, recent developments in luminescent materials are requiring a means to design advanced luminescence systems in which ionic and covalent bonding coexist, and systems in which inorganic and organic substances are mixed in nanostructures—How should we handle the electron–phonon coupling strength?

The influence of phonons sensed by electrons is mainly the electric field created by surrounding atoms, ions, and molecules. A simple way, supported by experimental results, is to describe the surrounding atoms, ions, and molecules as a sum of electric dipole moments. This electric dipole moment vibrates along with the vibration of the atoms, and as a result, an electric field that vibrates is generated. If you write the electric dipole moment as p and the electric field E, the E generated anywhere by p is proportional to p. On the other hand, the major electronic transition for Ln^3+^ ions is the electric dipole transition. If you write the transition dipole moment as M, the Hamiltonian of the interaction can be written as

(3) H=−M·E∝−M·p.

When this interaction is treated in perturbation theory, the transition rates of phonon absorption and emission are given as [2]

(4) $$W_{\mathrm{nr}}^{\mathrm{ab}}=\frac{3\omega_{fi}^{3}}{2\pi \rho v^{5}\hbar }{\left|\langle \psi_{f}^{\mathrm{el}}|-M\frac{ep}{4\pi \varepsilon R^{3}}|\psi_{i}^{\mathrm{el}}\rangle \right|}^{2}n\left(\hbar \omega_{fi}\right),$$

(5) $$W_{\mathrm{nr}}^{\mathrm{em}}=\frac{3\omega_{fi}^{3}}{2\pi \rho v^{5}\hbar }{\left|\langle \psi_{f}^{\mathrm{el}}|-M\frac{ep}{4\pi \varepsilon R^{3}}|\psi_{i}^{\mathrm{el}}\rangle \right|}^{2}\left(n\left(\hbar \omega_{fi}\right)+1\right).$$

Fortunately, the shift between the ground state and the excited state of the Ln^3+^ ion in the configuration coordinates is smaller than that of the transition metal ion, organic molecule, and semiconductor. The treatment of organic molecules is somewhat complicated, but the qualitative argument is not impaired. The theoretical treatment of heat emission in organic dyes has been actively reported since the 1960s [7,8,9,10,11]. However, simple guidelines and knowledge that can be applied in material design have not been obtained.

**Figure 1:**
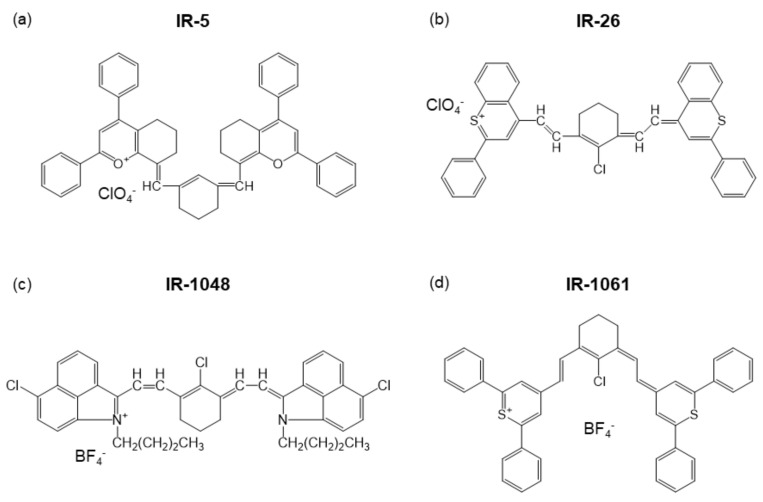
Examples of NIR laser dyes, (**a**) IR-5, (**b**) IR-26, (**c**) IR-1048, and (**d**) IR-1061.

Looking at the catalog for dye lasers [12], many of the NIR fluorescent dyes use ${\mathrm{ClO}}_{4}^{-}$, a slightly unfamiliar inorganic molecular anion, to neutralize the charge of organic cations, as illustrated in Figure 1a,b. During the 1980s, a vast number of organic fluorescent dyes that emit NIR fluorescence were developed for the purpose of optical amplification in fiber optical communication [13,14,15,16,17,18,19,20,21]. The properties of these NIR fluorescent dyes, including counter ions, maximum emission wavelength, λ_max_, and solvent for the dyes, are summarized in Table 1. As emphasized in Table 1, ${\mathrm{BF}}_{4}^{-}$ is used as a counter anion in IR-1040, IR-1048, and IR-1061 (Figure 1c,d). Among them, IR-1061 has been embedded in a variety of nanostructures such as polymeric micelles and solid nanoparticles and used for biophotonic applications [22,23,24,25,26,27,28]. Thinking about why such heavy anions are coordinated with the NIR organic dyes gives a hint on selecting proper surroundings in organic/inorganic hybrid nanostructures.

First, we start with a discussion of ℏω. When the effective mass that forms the vibration is μ and the bond strength (spring constant) is k, ω is expressed as

(6) $$\omega =\sqrt{k/\mu }.$$

Accordingly, the heavier the constituent ions, the smaller the phonon energy and the better for suppressing the phonon emission. In chloride, generally the multiphonon relaxation is suppressed more than in fluoride. The mass numbers of fluorine and chlorine are around 19 and 35, respectively, whereas the molecular masses of the ${\mathrm{BF}}_{4}^{-}$ and ${\mathrm{ClO}}_{4}^{-}$ are overwhelmingly heavier at around 87 and 100, respectively. Furthermore, since the distance between the charge centers is large from the viewpoint of ionic radius, it is estimated that the Coulomb force is weaker, and accordingly, *k* is smaller for the ${\mathrm{BF}}_{4}^{-}$ and ${\mathrm{ClO}}_{4}^{-}$. Within the molecules of ${\mathrm{BF}}_{4}^{-}$ and ${\mathrm{ClO}}_{4}^{-}$, there seems to be ionic oscillations, but these molecules have strong covalent bonds with the ${\mathrm{sp}}^{3}$ hybrid orbital. In a covalent bond, since the electron exists at the center of the bond, the vectors from the atom to the electron cancel each other out, and the electric dipole moment becomes small. Looking at the catalog carefully [12], all the solvents for NIR-emitting laser dyes are highly hydrophobic; that is, they are solvents with low polarity.

To control heat absorption and emission, regardless of whether it is organic or inorganic, we should pay attention to the energy of vibration, ℏω, and the polarity of the space created by the atoms, ions, and molecules surrounding the luminescence center. The smaller the vibration energy and polarity, the less heat will be emitted. In the following sections, we discuss the application of nanostructures in which ions and molecules are hybridized and designed for NIR biomedical photonics based on these concepts.

## 2. Dye-Loaded Nanostructure for NIR Fluorescence

### 2.1. Biomedical Probe Design Using Organic Molecules

Along with advances in imaging instruments that can efficiently detect over-thousand nanometer (OTN)-NIR light, NIR organic fluorophores are facilitating biomedical imaging—from contrast-enhanced imaging of anatomical structures and molecular imaging of specific biomarkers to functional imaging of physiological activities [32]. Small organic fluorophores are suitable for their rapid urinal excretion and flexible structures that can be altered via conjugation methods for targeting biomolecules. A series of organic fluorophores developed for the NIR-I (650–950 nm) window include indocyanine green (ICG; peak emission at 830 nm) [33], methylene blue (MB; peak emission at 686 nm) [34], C700-X and C800-X families [35,36], and boron–dipyrromethene (BODIPY) [37]. On the other hand, OTN-NIR organic dyes are classified into three major types: organic dyes with benzobisthiadiazole (BBTD) acceptors, aggregation-induced emission fluorogens (AIEgens), and polymethine cyanine dyes [38]. A notable donor–acceptor–donor (D–A–D) organic molecule with BBTD is CH1055, whose derivatives are demonstrated for various OTN-NIR biomedical applications [39,40,41]. Imaging of blood flow disturbance in the brain through the skull has also been reported using a D–A–D organic dye, IR-E1, in mice [42]. AIEgens, mostly composed of a D–A–D scaffold with a BBTD acceptor core, exhibit stronger NIR emission with an increase in dye concentration through aggregation, and representative examples of the OTN-NIR AIEgens are TQ-BPN, TB1, and HLZ-BTED [43,44,45]. Polymethine fluorophores are one of the most significant classes of OTN-NIR organic dyes, including commercially available IR-1048 and IR-1061 [31], flavylium polymethine dye (Flav7) [46], and Flav7 analog dye (FD-1080) [47]. Among these, IR-1061 is a major and famous OTN-NIR fluorescent dye of particular interest in bioimaging, with deeper penetration (around 5–20 mm). Recently, the use of well-designed probes with IR-1061 for visualization of blood vessels and cancer lesions through the skin in mice has been increasingly reported, as described in later parts (see Section 2.2 and Section 2.3). IR-1061 consists of a cationic backbone and a nonpolar counter ion, ${\mathrm{BF}}_{4}^{-}$. IR-1061 is a molecular dye that shows high quantum yield but is poorly soluble in water and easily quenches by replacing ${\mathrm{BF}}_{4}^{-}$ with polar hydroxyl ions when exposed to aqueous and physiological environments. The hydroxyl ion only has a small mass but a large phonon energy with polar vibration that can serve as a “wicked witch”. Therefore, methods have been investigated for maintaining the OTN-NIR fluorescence of IR-1061 by encapsulating the dye in a hydrophobic microenvironment in core-shell nanoparticles for better use in biomedical fields [25,27,48,49].

Nanoparticles are generally defined as materials with a size of 1–100 nm and are important in nanotechnology because of their specific physicochemical properties and biological effects compared with larger particles [50]. Especially in the biomedical field, nanoparticles are useful as carriers that protect encapsulated and loaded molecules from the external environment and that transport and release them into target tissues and organs [51]. As drug delivery systems, nanoparticles of polymeric micelles and liposomes encapsulating drug molecules can control the distribution of drugs in the body [52], thereby reducing side effects and enhancing drug efficacy [53]. In 2020, nanoparticle-type vaccine was developed to gain effective immune induction by encapsulating mRNA with low in vivo stability in lipid nanoparticles for preventing coronavirus disease 2019 (COVID-19), which caused a global pandemic, [54]. Among the nanoparticles having hydrophilic surface to stabilize existence in vivo, micellar nanoparticles can encapsulate poorly soluble molecules in their hydrophobic core [55]. Stimuli-sensitive polymer micelles have also been investigated as a carrier for drug delivery in chemotherapy for cancer [55]. In the field of bioimaging, nanoparticles with contrast capability in magnetic resonance and fluorescence have been studied to obtain qualitative and quantitative spatio-temporal information on intracellular and in vivo structures. Recently, the composition and design methods of core-shell nanoparticles have been investigated to maintain high fluorescence performance of IR-1061 encapsulated in a hydrophobic core.

### 2.2. Application of Solubility Parameters for Designing NIR Fluorescent Dye-Loaded Polymeric Micelles

**Figure 2:**
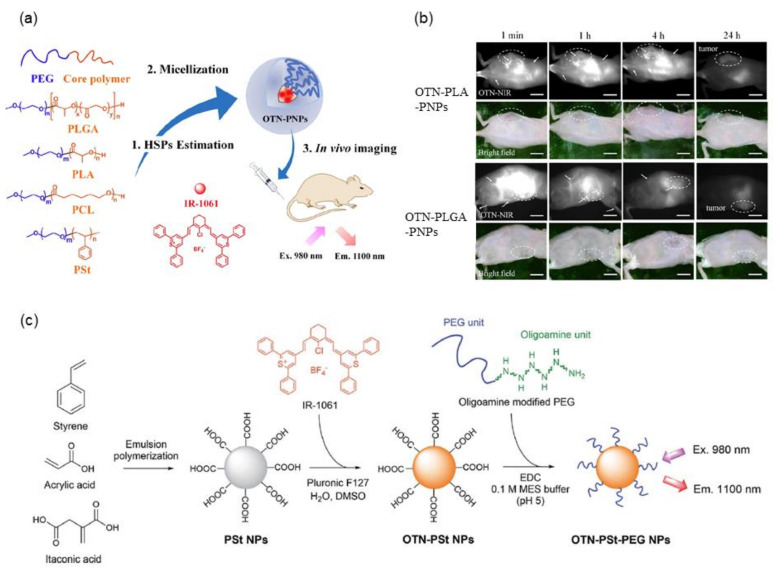
NIR dye-loaded luminescent polymeric micelles and solid nanoparticles. (**a**) Molecular structures of the compounds used for preparing OTN-polymeric nanoparticles (PNPs) and their schematics (Ex.: excitation; Em.: emission). (**b**) OTN-NIR fluorescence in vivo imaging of live mouse. OTN-PLA-PNPs (1st and 2nd row) and OTN-PLGA-PNPs (3rd and 4th row) dispersed in PBS were injected intravenously into female BALB/c mice inoculated with colon-26 cells (Scale bar, 10 mm). (**c**) Schematics of NIR fluorescent dye-loaded polystyrene (PSt) nanoparticles. (**a**,**b**) are adapted from reference [57] and (**c**) from reference [28] with permission with the terms of the Creative Commons CC BY license.

Micellar nanoparticles of various block copolymers such as poly(ethylene glycol) (PEG)-*block*-poly(lactic-co-glycolic acid) (PLGA), PEG-*block*-poly(lactic acid) (PLA), PEG-*block*-polycaprolactone (PCL), and PEG-*block*-polystyrene (PSt) have been widely used for encapsulating hydrophobic drug molecules [56]. For designing highly fluorescent nanoparticles encapsulating IR-1061, a quantitative evaluation of the affinity of the dye molecule and the core polymer is useful, which can be estimated using solubility parameters. Previous studies have demonstrated that matching the solubility parameters of dyes and core polymers realizes the design of high-performance fluorescent polymeric micellar nanoparticles containing hydrophobic dyes [57], as shown in Figure 2a. The Hansen solubility parameter (HSP) is described by three components—dispersion, polarity, and hydrogen bonding separately [58,59] (see Section 3 for details)—and thus allows quantitative consideration of the polarity of molecules. While the HSPs of polymers, such as PLGA, PLA, PCL, and PSt, that can form micelle cores have been reported in the published literature [60,61,62,63], they can be calculated by the molecular group contribution method [64] based on their chemical structure. The HSPs of dye molecules, even if they are an ionic compound such as IR-1061, can be calculated by the Hansen solubility sphere method [65] from the results of solubility tests in various organic solvents. When a micellar nanoparticle is composed of a polymer core having a chemical structure with a small energy difference from the dye molecule IR-1061, based on the three factors of dispersion, polarity, and hydrogen bonding, the nanoparticle encapsulates the dye with high efficiency and provides a probe with high fluorescence performance and encapsulation stability [57]. Note that the increased affinity between the dye and the polymer prevents the invasion of water molecules that can couple with IR-1061 as a quencher into the hydrophobic core of polymer micelles. This results in improved stability of the fluorescent polymer micelles in aqueous and physiological environments. The polarity term mostly contributes to the affinity between the polymers and the dye. As shown in Figure 2b, stable fluorescent nanoparticles containing IR-1061 show long circulation (several hours) in the blood and provide contrasts on cancer lesions [57], possibly via the enhanced permeability and retention effect [66]. Modification of the core structure of PEG-*block*-PCL micellar nanoparticles also altered their stability in an in-vivo environment. Introducing benzyl carboxylate into the core PCL enhanced the stability and imaging performance for cancer lesions [67]. Overall, the affinity of the core polymer and dye molecules, mainly dominated by the difference in their polarities, can be evaluated by the solubility parameters, HSPs. Stable and biocompatible in vivo imaging contrast agents with high fluorescence performance and stability can be developed by matching the HSPs of the polymer and dye molecules.

### 2.3. Designing Dye-Loaded Solid Polymer Nanoparticles by Adjusting the Polarity of Polymers

The adjustment of the polarity of a core polymer with NIR fluorescent dye molecules is useful not only in designing polymeric micellar nanoparticles but also in the design of NIR fluorescent probes using solid nanoparticles. A recent study using copolymer of styrene and acrylic acid monomer units has demonstrated the effect of the core polarity, which is adjusted by the acrylic acid ratio, on the molecular state of IR-1061 encapsulated in the polymer core [28], as schematically shown in Figure 2c.

Styrene is a hydrophobic and less polar monomer, while acrylic acid is a water-soluble and highly polar monomer with carboxyl groups. Therefore, the polarity of the PSt core can be controlled by changing the ratio of acrylic acid introduced into the PSt. IR-1061 can be introduced in the PSt-based solid nanoparticles (diameter: approximately 50 nm) in the synthetic process via emulsion polymerization. The amount of OTN-NIR fluorescent dye IR-1061 encapsulated and the fluorescent performance of the resulting dye-encapsulated PSt-based nanoparticles are positively correlated with the acrylic acid ratio, i.e., the introduced polarity, of the PSt-based particle core [28]. A hydrophilic shell layer is obtained by chemical modification of the acrylic acid unit in the polymer that forms the nanoparticle core, via covalently attaching PEG with an oligoamine at one end. These OTN-NIR fluorescent dye-loaded PEGylated polystyrene-based nanoparticles have low cytotoxicity and high dispersion stability under physiological conditions containing serum proteins. The designed OTN-NIR fluorescent polystyrene nanoparticles are also applicable for angiography for several hours after intravenous injection in mice [28].

The series of findings described above indicate that the amount of dye encapsulation and fluorescent performance of nanoparticle probes can be enhanced by optimizing the polarity of the particle core. Additionally, the resulting OTN-NIR fluorescent particles are useful as in vivo imaging contrast agents.

## 3. Organic/Inorganic Hybrid Nanostructure for NIR Fluorescence

### 3.1. Molecular Upconversion Phosphors: Erbium (III) Complex Coordinated with $BF_{4}^{-}$ Ions

**Figure 3:**
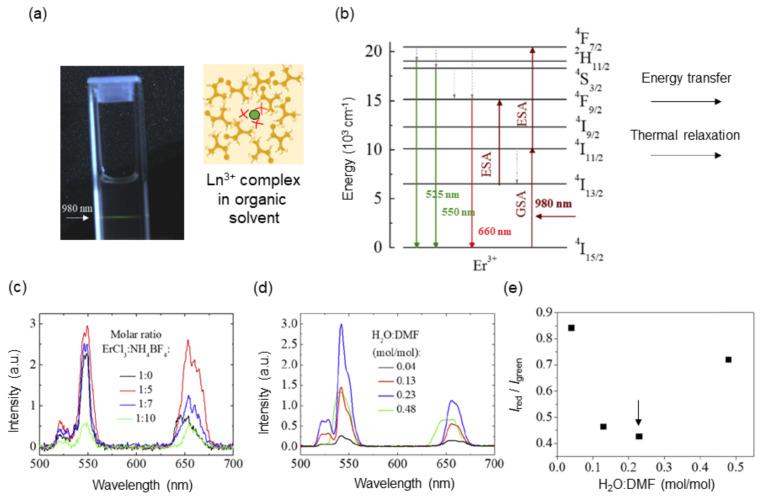
Upconversion luminescence from Er^3+^ complexes coordinated with ${\mathrm{BF}}_{4}^{-}$ anions under 980 nm irradiation. (**a**) A picture of the Er^3+^ complexes (ErCl_3_/NH_4_BF_4_ = 1:5) in a cuvette and schematic of Er^3+^ coordination complex in organic solvent. (**b**) Energy level diagram of Er^3+^. (**c**) Upconversion spectra of the Er^3+^coordination complex with different molar ratio of ErCl_3_/NH_4_BF_4_ (power: 192 W/cm^2^, solvent: DMF). (**d**) Upconversion spectra of the Er^3+^ coordination complexes dispersed in the mixture of H_2_O and DMF at different mixed ratios. (**e**) Red-to-green emission intensity ratio (*I*_red_/*I*_green_) as a function of the solvent mixture ratio. *I*_red_ and *I*_green_ correspond to integrated emission intensity in the ranges of 615–700 nm and 500–575 nm, respectively. An arrow indicates an optimum solvent mixture ratio that allows for the least amount of thermal relaxation among all. Adapted from reference [73] with permission. Copyright 2020 Optical Society of America under the terms of the OSA Open Access Publishing Agreement.

NIR organic laser dyes such as IR-1061 are coordinated with nonpolar ${\mathrm{BF}}_{4}^{-}$ anions for reducing nonradiative relaxation, which is heat release upon optical excitation. The coordination of such heavy and large anions to the luminescence center can be applied to the design of Ln^3+^-based molecular upconversion luminescent phosphors. To date, molecular upconversion fluorophores have been reported on the basis of organic ligands [68,69,70] and metal–organic frameworks (MOFs) [71,72]. The thermal quenching of upconversion luminescence is mostly originated in high-energy OH oscillators, and thus these molecular systems are realized by stabilizing the coordination of compounds in water-free polymeric hosts with high hydrophobicity. Our group has demonstrated an upconversion luminescent complex based on Ln^3+^ coordinated by ${\mathrm{BF}}_{4}^{-}$ tetrahedrons [73], as shown in Figure 3. The complexes were synthesized by the introduction of tetrafluoroborate salts (NH_4_BF_4_ and NaBF_4_) to lanthanide chlorides (ErCl_3_ and EuCl_3_) in N,N-dimethylformamide (DMF). The Cl^–^ ligands were replaced by the ${\mathrm{BF}}_{4}^{-}$ ligands for Er^3+^ in the luminescent systems, and they were removed as a precipitate of either NH_4_Cl or NaCl, which was confirmed by X-ray diffraction analysis. The systematic spectroscopic study revealed that the highest emission intensity, lower red-to-green fluorescence ratio *I*_red_/*I*_green_ (*I*_red_: 630–680 nm; *I*_green_: 509–576 nm) and narrower emission lines were realized at a mixed molar ratio of Er^3+^:${\mathrm{BF}}_{4}^{-}$ = 1:7 and in solvents with molar ratio of H_2_O/DMF = 0.23 (Figure 3c–e). The emission properties of the Er^3+^ complex suggested that the nonlinear upconversion luminescence was achieved because ${\mathrm{BF}}_{4}^{-}$ ligands occupied the first coordination sphere and hampered the thermal quenching. As shown in Figure 3b, a nonradiative decay, which is heat emission, derived from transitions ^4^S_3/2_→^4^F_9/2_ and ^4^I_11/2_→^4^I_13/2_ corresponds to an increase in the *I*_red_/*I*_green_, indicating that the lower *I*_red_/*I*_green_ obtained means more suppression of heat emission (thermal relaxation) in the luminescent system. This decay is described by the multiphonon relaxation rate, *W*_MPR_, in Equation (1).

As partially demonstrated by the change in luminescent intensity at different mixed ratio of H_2_O and DMF, the chemical polarity of solvents strongly affects luminescence properties such as emission intensity, spectral shape, and lifetime. The polarity of molecules is quantified by the HSP [58,59]. This solubility parameter comprises a summation of the cohesive energy density of a solvent, including all of the intermolecular attraction in liquid: dispersion, polar, and hydrogen bonding. This is written in terms of the solubility parameter *δ*, in the form

(7) $$\delta_{T}^{2}=\delta_{d}^{2}+\delta_{p}^{2}+\delta_{h}^{2}$$

where *δ*_T_, *δ*_d_, *δ*_p_, and *δ*_h_ are the solubility parameters that correspond to the total, dispersion, polar, and hydrogen bonding, respectively [74]. The HSPs, which help to estimate miscibility, adhesion, and wetting, are mainly used to guide organic solvent selection, salt screening, and solid dispersion in pharmaceutic fields [75]. Based on the discussion of the relationship between luminescence intensity and polarity of surrounding molecules, solvents with a smaller polarity are in general suitable for the Ln^3+^ luminescent complex. Interestingly, however, the upconversion luminescence intensity increased with an increase in water concentration up to a certain hydration level (Figure 3e), suggesting that OH oscillators did not linearly contribute to the nonradiative relaxation of Er^3+^ excited states. This result implied that there might be a more favorable ionic environment that hampers the direct coordination of water to Ln^3+^ ions.

### 3.2. Application of Solubility Parameters for the Design of Ln^3+^-Containing Organic/Inorganic Hybrid Nanoprobes

**Figure 4:**
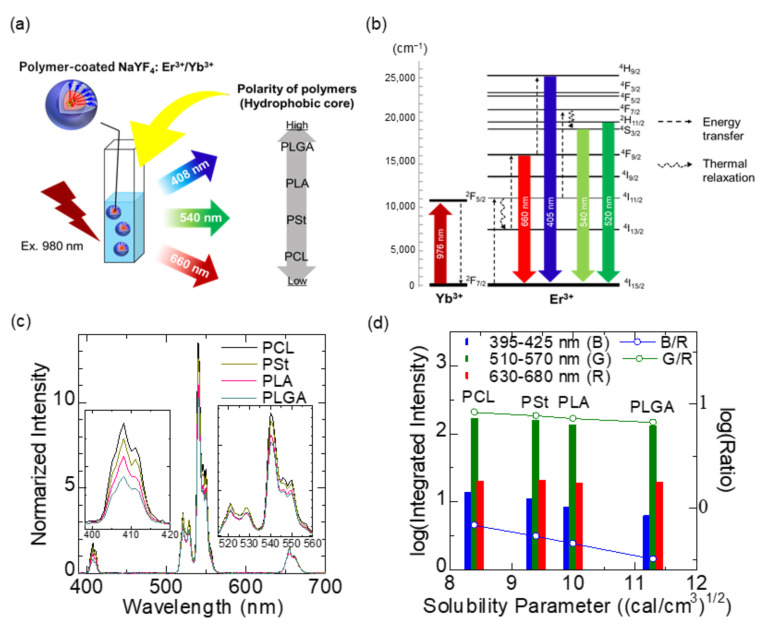
Effect of hydrophobic polymer on upconversion emission of polymer-coated NaYF_4_:Yb^3+^,Er^3+^ nanoparticles dispersed in water. (**a**) Illustration of the samples and the order of chemical polarities of the polymers. (**b**) Energy level diagram of NaYF_4_:Yb^3+^,Er^3+^. (**c**) Upconversion spectra of polymer-coated NaYF_4_:Yb^3+^,Er^3+^ with different hydrophobic core polymers. Each spectrum shown is normalized with a peak height at 660 nm. (**d**) Integrated intensity of the upconversion emission of each wavelength range and their ratio to the intensity of red (630–680 nm). The relationship between the emission intensities and the ratio with the solubility parameter of the hydrophobic core polymers is shown. Adapted from reference [83] with permission. Copyright 2021 Elsevier B.V.

Control over the electric dipole moment of surroundings, such as solvent and anions, around the luminescence center is one of the foremost aspects in the design of Ln^3+^-based luminescence nanosystems. The influence of surrounding atoms, ions, and molecules on the electronic transition of Ln^3+^ has an interaction length of >10 nm [76], which is not necessarily a crucial problem in the development of bulk solid-state phosphors. However, for nanoparticles of roughly 1–50 nm in diameter, the thermal emission of the electron system is affected not only by the nanosized crystalline host but also by solvent molecules outside the host nanoparticles. Ln^3+^-doped inorganic nanocrystals are recognized as stable and efficient phosphors. Sodium yttrium fluoride (NaYF_4_), for example, is a suitable host lattice for visible and NIR luminescence due to its low phonon energy (~360 cm^–1^) and for precise size controllability down to single nanometers [77,78,79]. For maintaining the luminescence properties of Ln^3+^-doped nanocrystals in physiological condition, surface coating by hydrophobic polymers [80] and ceramics shell formation [81] are usually performed, followed by surface modification using biocompatible PEG block copolymer [82]. Both approaches are intended to shield Ln^3+^ ions from stretching vibrations of surrounding molecules. Simply put, surrounding Ln^3+^ with either ionic crystals with low phonon energy or molecules with less chemical polarity is the key to the realization of bright illumination from Ln^3+^-doped nanoparticles. Nevertheless, few studies have been performed to systematically evaluate the effects of surroundings on the luminescence properties of Ln^3+^-doped nanoparticles, especially by the chemical polarity. We therefore investigated the effects of dispersion solvents and coating polymers on the upconversion luminescence of NaYF_4_:Yb^3+^,Er^3+^ nanoparticles under 980 nm optical excitation [83], as shown in Figure 4a,b. Spectroscopic measurements were performed on the following luminescence systems: (1) NaYF_4_:Yb^3+^,Er^3+^ nanoparticles dispersed in solvents such as dichloromethane, tetrahydrofuran, cyclohexane, and hexane and (2) water dispersion of NaYF_4_:Yb^3+^,Er^3+^ nanoparticles coated with either PCL, PSt, PLA, or PLGA as a hydrophobic part of the PEG block copolymer. Table 2 summarizes the HSPs of solvents and polymers used, as well as of blood components [60,62,63,84,85,86]. Note that the total solubility parameter, δ_T_, of PLGA varies in the range of 10.3–11.3 depending on the ratio of lactide-to-glycolide [62]. The results obtained from the system (1) revealed that the upconversion emission spectra, such as the green and blue emission (540 and 408 nm, respectively) ratios to the red (660 nm), were well correlated with the polarity of the molecules that surround the nanoparticles. In the system (2), the intensity of 408 and 540 nm was highest when the NaYF_4_:Yb^3+^,Er^3+^ nanoparticles were coated by PCL, which has the lowest polarity among the four polymers, whereas the one of 660 nm was highest when the nanoparticles were coated by PLGA having the highest polarity (Figure 4c,d). This is because the thermal relaxation (^4^I_11/2_ → ^4^I_13/2_) was enhanced with an increase in the polarity, resulting in increased emissions at 660 nm (^4^F_9/2_ → ^4^I_15/2_) and decreased emissions at 405 nm (^4^H_9/2_ → ^4^I_15/2_). The results suggested that when polymers and solvents possess a high solubility parameter, the degree of thermal quenching tends to increase.

**Figure 5:**
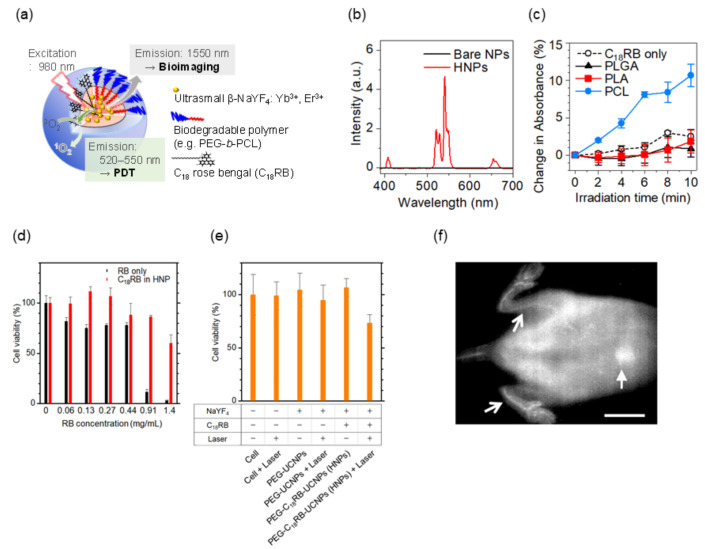
Upconversion luminescent nanostructure for NIR-induced photodynamic therapy. (**a**) Design of hybrid nanoprobes (HNPs) composed of hydrocarbonized photosensitizer (C_18_ rose bengal dye, RB) and upconversion ultrasmall NaYF_4_:Er^3+^,Yb^3+^ nanoparticles (NPs). At the hydrophobic core of biocompatible PEG-b-PCL copolymers, the dyes are coupled with a cluster of the upconversion nanoparticles. Under 980 nm irradiation, the nanoparticles emit upconversion visible luminescence (520–550 nm) that activates C_18_ RB dyes to generate singlet oxygen for photodynamic therapy and 1550 nm NIR luminescence for in vivo bioimaging. (**b**) Upconversion luminescence spectra of the HNPs dispersed in water. Bare NaYF_4_:Er^3+^,Yb^3+^ NPs were used as a control. (**c**) Generation of singlet oxygen by HNPs (1 mg/mL) in water under 980 nm light irradiation (0.8 W/cm^2^). (**d**) Cytotoxicity of free RB and C_18_ RB-loaded HNPs on cultured Colon-26 cells without 980 nm optical excitation. The toxicity of the RB compounds was reduced by the encapsulation. (**e**) NIR-induced photodynamic therapy effect of HNPs on Colon-26 cells. (**f**) NIR fluorescence in vivo imaging with the HNPs in mice (wavelength: 1550 nm; Scale bar: 10 mm). Arrows and a block arrow indicate hindlimb blood vessels and liver, respectively. Adapted from reference [87] with permission with the terms of the Creative Commons CC BY license.

Based on this systematic study, PEG-*block*-PCL copolymer was proved to be preferable because it provides the Ln^3+^-doped nanoparticles with biocompatibility and strong luminescence in water. Note that hydrophobic organic dyes retain their luminescence property and are stable when surrounded by solvents or molecules with a smaller polarity. With this in mind, we designed a biodegradable, organic/inorganic hybrid nanostructure for NIR-induced photodynamic therapy using ultrasmall NaYF_4_:Yb^3+^,Er^3+^ nanoparticles (~9 nm in diameter) and hydrocarbonized rose bengal dyes, both of which were encapsulated at the core of PEG-*block*-PCL micelles [87]. Since Ln^3+^-doped nanoparticles emit both upconversion visible light for photoactivating the dyes and NIR light for in vivo fluorescence imaging, this probe can be useful for tracing the biodistribution of nanoparticles during photodynamic therapy, as shown in Figure 5a,b. The hydrophobic part of hydrocarbonized rose bengal dyes worked well when involved in the PCL core and located the dyes on the surface of the PCL core, resulting in efficient absorption of green light and the emission of singlet oxygen to surrounding water possible. By encapsulation of the dyes and Ln^3+^-doped nanoparticles in the hydrophobic PCL core, the green upconversion luminescence was effectively enhanced and the increased activation of the rose bengal dyes led to larger amount of singlet-oxygen generation (Figure 5c–e). In addition, the hybrid nanostructures developed can be degradable after their use in vivo because the size of the nanoparticles allows their renal excretion and because the PEG-*block*-PCL polymers are biodegradable. The hybrid nanoparticles administered in mice were visualized by 1550 nm NIR luminescence in deep tissue, showing the possibility of visualizing blood and organs, (Figure 5f) although further development is needed to realize simultaneous photodynamic therapy and in vivo imaging. The studies that are described in this section pave the way to the effective use of HSPs for selecting proper biomaterials in Ln^3+^-containing nanosystems.

## 4. Conclusions

Heat emission in a photonic system that involves near infrared (NIR) light is intense because the energy level intervals used for electronic transitions are narrow, as compared to those of visible light. Therefore, the suppression of heat emission in luminescence materials is of considerable interest, particularly with regard to the spectral property of nanosized phosphors. Furthermore, recent developments in NIR-emitting dyes and nanoparticles require new material design guidelines that will allow organic/inorganic hybrid structures for biomedical fields. Taking the property of materials that surround luminescence centers such as molecules and ions into account, the use of a system composed of heavy elements and formed by weak bonds is a reasonable choice due to the efficient suppression of phonon energy.

This review proposes that the polarity and vibrational energy of the surroundings have a dominant effect on the luminescence systems. We introduced NIR photoexcited phosphors and nanostructures for photodynamic therapy and organic/inorganic mixed systems designed based on the following guidelines: heavy elements, weak bonds, and weak polarities. These guidelines for the design of organic/inorganic hybrid nanostructures would accelerate the development of biomedical photonics, which has become increasingly important in recent years.

## Figures and Tables

**Table 1:** Properties of NIR laser dyes coordinated with a counter ion. The counter ions are emphasized in the compound name.

Product Name	Compound Name	Counter Ion	λ_max_	Solvent	Reference
IR-5	8-[[3-[(6,7-dihydro-2,4-diphenyl-5H-1-benzopyran-8-yl)methylene]-1-cyclohexen-1-yl]methylene]-5,6,7,8-tetrahydro-2,4-diphenyl-1-Benzopyrylium **perchlorate**	${\mathrm{ClO}}_{4}^{-}$	1320	Dichloroethane	[29]
IR-26	4-(7-(2-phenyl-4H-1-benzothiopyran-4-ylidene)-4-chloro-3,5-trimethylene-1,3,5-heptatrienyl)-2-phenyl-1-benzothiopyrylium **perchlorate**	${\mathrm{ClO}}_{4}^{-}$	1180	Dichloroethane	[30]
IR-132	2-[2-[2-(diphenylamino)-3-[2-[3-(4-methoxy-4-oxobutyl)naphtho [2,3-d]thiazol-2(3H)-ylidene]ethylidene]-1-cyclopenten-1-yl]ethenyl]-3-(4-methoxy-4-oxobutyl)-Naphtho[2,3-d]thiazolium **perchlorate**	${\mathrm{ClO}}_{4}^{-}$	972	Dimethyl sulfoxide	[20]
IR-140	5-chloro-2-[2-[3-[(5-chloro-3-ethyl-2(3H)-benzothiazol- ylidene)ethylidene]-2-(diphenylamino)-1-cyclopenten- 1-yl]ethenyl]-3-ethyl benzothiazolium **perchlorate**	${\mathrm{ClO}}_{4}^{-}$	910	Dimethyl sulfoxide	[19]
IR-1040	4-[2-[3-[(2,6-diphenyl-4H-thiopyran-4-ylidene)ethylydene]-2-phenyl-1-cyclohexen-1-yl]ethenyl]-2,6-diphenyl-thiopyrilium **tetrafluoroborate**	${\mathrm{BF}}_{4}^{-}$	1040	Dichloromethane	[31]
IR-1048	1-butyl-2-[2-[3-[2-(1-butyl-6-chlorobenz[cd]indol-2(1H)-ylidene)ethylidene]-2-chloro-1-cyclohexen-1-yl]ethenyl]-6-chloro-benz[cd]indolium **tetrafluoroborate**	${\mathrm{BF}}_{4}^{-}$	1048	Dichloromethane	[31]
IR-1061	4-[2-[2-Chloro-3-[(2,6-diphenyl-4H-thiopyran-4-ylidene)ethylidene]-1-cyclohexen-1-yl]ethenyl]-2,6-diphenyl-thiopyrylium **tetrafluoroborate**	${\mathrm{BF}}_{4}^{-}$	1061	Dichloromethane	[31]

**Table 2:** Hansen solubility parameters (HSPs) of solvents and polymers used in the design of Ln^3+^-containing nanomaterials [28,73,83,87] and of blood components [85]. The data are adapted from references [60,62,63,84,85] with permission.

Name	Hansen Solubility Parameter (cal/cm^3^)^1/2^				Reference
	δ_d_	δ_p_	δ_h_	δ_T_	
n-Hexane	7.3	0.0	0.0	7.3	[84]
Cyclohexane	8.2	0.0	0.1	8.2	[84]
Tetrahydrofuran	8.2	2.8	3.9	9.5	[84]
Dichloromethane	8.9	3.1	3.0	9.9	[84]
N,N-dimethylforamide (DMF)	8.5	6.7	5.5	12.1	[84]
Water	7.6	7.8	20.7	23.4	[84]
Poly-ε-caprolactone (PCL)	7.8	0.7	1.0	7.9	[63]
Polystyrene (PSt)	9.4	0.4	1.0	9.4	[60]
Poly(L-lactide) (PLA)	9.7	2.0	3.3	10.4	[63]
Poly(D,L-lactide-co-glycolide) (PLGA) ^1.^	8.0	5.2	6.0	11.3	[62]
Poly(acrylic acid) (PAA)	–	–	–	11.4	[86]
Polyethylene glycol (PEG)	9.9	4.7	2.9	11.4	[63]
Cholesterol and esters	–	–	–	8.4–8.6	[85]
Triglycerides	–	–	–	8.0–8.3	[85]
Lipid-soluble vitamins	–	–	–	8.6–10.9	[85]
Phospholipids	–	–	–	>16	[85]
Non-denatured proteins	–	–	–	>18	[85]

^1.^ Lactide:Glycolide = 50:50.
